# EPA and DHA Fatty Acids Induce a Remodeling of Tumor Vasculature and Potentiate Docetaxel Activity

**DOI:** 10.3390/ijms21144965

**Published:** 2020-07-14

**Authors:** Caroline Goupille, Sophie Vibet, Philippe G. Frank, Karine Mahéo

**Affiliations:** 1 Laboratoire Nutrition, Croissance et Cancer, N2C UMR 1069, University of Tours, INSERM, F-37032 Tours, France; caroline.goupille@univ-tours.fr (C.G.); sophie.vibet@gmail.com (S.V.); Philippe.Frank@univ-tours.fr (P.G.F.); 2 Service gynécologie, CHRU (Centre Hospitalier Régional Universitaire) de Tours, Hôpital “Bretonneau”, F-37044 Tours CEDEX 09, France; 3 Laboratoire de Physiologie, Faculté de Pharmacie, F-37200 Tours, France

**Keywords:** n-3 PUFA, docetaxel, vascularization, epiregulin, amphiregulin, mammary tumors

## Abstract

n-3 long chain Polyunsaturated Fatty Acids (n-3 LCPUFA) have been shown to improve the efficacy of conventional chemotherapies used for breast cancer treatment. In addition to their reported ability to increase the chemosensitivity of cancer cells, we hypothesized that n-3 LCPUFA could induce a remodeling of the vascular network in mammary tumors. A contrast-enhanced ultrasound method was used to monitor the vascular architecture during docetaxel treatment of mammary tumors in rats fed either a control or an n-3 LCPUFA-enriched diet (docosahexaenoic acid (DHA)/eicosapentaenoic acid (EPA)). The vascular network was remodeled in favor of smaller vessels (microvascularization), which represented 54% of the vasculature in n-3 LCPUFA tumors but only 26% in control tumors after 2 weeks of chemotherapy. Importantly, vascularization changes occurred both before and during docetaxel treatment. The density of smaller vessels quantified before chemotherapy was correlated with improved tumor size reduction by docetaxel treatment. Furthermore, transcript levels of the angiogenesis-specific genes epiregulin and amphiregulin were reduced by ~4.5- and twofold in tumors obtained from rats fed an n-3 LCPUFA-enriched diet compared to those of rats fed a control diet, respectively. Their expression levels were negatively correlated with tumor regression after chemotherapy. Taken together, this preclinical data strengthen the potential usefulness of n-3 LCPUFA as a complementary clinical strategy to improve drug efficiency via remodeling of the tumor vasculature.

## 1. Introduction

Unlike the normal vascular network, the tumor vascular tree lacks an orderly branched hierarchy from large vessels to successively smaller vessels (microvascularization) that normally feed a capillary bed involved in the exchange of molecules such as oxygen, nutrients, or therapeutic agents. In addition, tumor vasculature quality is admittedly poorly developed, with highly dilated and permeable vessels leading to high interstitial fluid pressure. These differences can irreversibly affect the delivery of molecules and lead to poorly perfused regions within the tumor [1]. These types of structural abnormalities of the tumor vasculature have far-reaching consequences that include poor response to anticancer therapies. Studies have identified an apparent conflict between the use of antiangiogenic molecules targeting tumor vascularization and adequate tumor perfusion for effective intravenous chemotherapeutic agent delivery. Several lines of evidence have indicated that antiangiogenic treatments can “normalize” an abnormal structure and function of the tumor vasculature. Vessels become smaller in diameter, less tortuous and are more efficient at delivering oxygen, nutrients, and drugs [2,3].

Using the ultrasound contrast agent SonoVue^®^ (microbubbles of sulfur hexafluoride), tumor functional vascularization, from large to small vessels, may be visualized and quantified by contrast-enhanced ultrasound (CEUS). Microbubbles, with a diameter equivalent or inferior to red blood cells (10 µm), circulate into capillaries and increase ultrasound signal detection. Larger vessels contained more contrast agents than the smaller ones. This system therefore permits a scaled intensity of contrast enhancement. We have previously reported an original contrast-enhanced ultrasound analysis method (CEUS) capable of discriminating and quantifying between vessels according to their diameters [4]. This method is based on discrimination of pixels according to their contrast intensity, which allows the quantification of macro-, medium- and microvascularization densities. This technique has been validated by standard immunohistological analysis and applied in a clinical setting to detect changes of the vascular architecture during chemotherapeutic treatment of human breast carcinoma [4].

Among n-3 long chain polyunsaturated fatty acids (n-3 LCPUFA), docosahexaenoic acid (DHA, 22:6n-3) and eicosapentaenoic acid (EPA, 20:5n-3) have generated an important interest due to their abilities to decrease chemotherapeutic resistance of experimental mammary tumors to anthracyclines, taxanes, or radiotherapy without additional side effects (reviewed in [5,6,7]). In addition to promoting an increased chemosensitivity of cancer cells, a nutritional supply of n-3 LCPUFA has been shown to act on the tumor microenvironment by inducing an antiangiogenic effect [8,9,10,11]. We previously reported that a reduction in the tumor vascularization during n-3 LCPUFA supplementation is accompanied by an enhancement of tumor sensitivity to anthracyclines or taxanes [8,12]. An n-3 LCPUFA diet can alter the tumor vasculature towards an increase in its functional efficacy (decreased interstitial fluid pressure and increased dye perfusion) [12].

Based on the hypothesis that functional modifications by n-3 LCPUFA may be associated with alterations in the vascular structure, the present study was set up to analyze the vascular tree and determine the ratio of macro-, medium- and microvascularization densities in a tumor model using CEUS methodology. Furthermore, the present study was performed to test the capacity of n-3 LCPUFA to induce a remodeling of the rat mammary tumor vascular architecture in favor of a microvascularization prior to or during chemotherapy with docetaxel, a major anticancer drug used in clinical practice. Additionally, by screening the transcription levels of genes involved in angiogenesis, this study identified n-3 LCPUFA-regulated genes, which expression levels correlate with vascular parameters and anti-tumor efficacy of docetaxel in rat mammary tumors.

## 2. Results

### 2.1. n-3 LCPUFA Supplementation Induces a Remodeling of the Mammary Tumor Vasculature Prior to the Initiation of Chemotherapy

**Figure 1:**
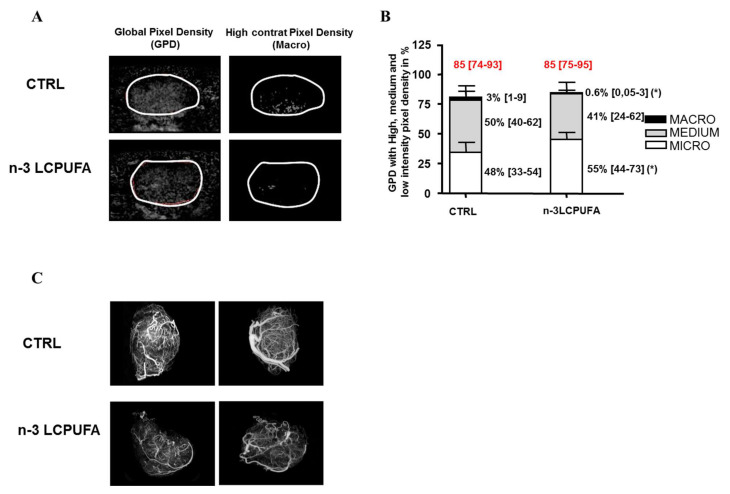
An n-3 long chain Polyunsaturated Fatty Acid (n-3 LCPUFA)-enriched diet induces a remodeling of the tumor vasculature in favor of the microvasculature. Female rats carrying mammary tumors were fed a control diet or an n-3 LCPUFA-enriched diet. Tumor vascularization was quantified using contrast-enhanced ultrasound (CEUS) methodology after SonoVue^®^ microbubbles injection when tumors reached 2 cm² (*n* = 14 by nutritional group). Using the MATLAB^®^ software a specific analysis of CEUS signal lead us to evaluate several vascular parameters within tumors defined as the region of interest (ROI). The global contrasted-pixel density (GPD) took into account all pixels with contrast signal and reflected a global vascular density. With discrimination of pixels according to their contrast signal intensity, three vascular compartments (macro-, medium- and micro-vasculature) were quantified and expressed as a proportion (%) of the global vascular network. (**A**) Representative images obtained by CEUS showing GPD reflecting global vascular density left panels) and macrovascularization (right panels). Contours of tumors were surrounded by the white line (ROI). Magnification factor: 1.5×. (**B**) Histograms representing the GPD, global vascular density (with its quantification in red), subdivided in the three compartments (micro, medium and macrovasculature) (bar and values on the right: median and interquartile range, 25–75th percentile) and obtained in mammary tumors of rat fed a control or n-3 LCPUFA diet (*n* = 14 per nutritional group). (**C**) Three-dimensional images of tumor vasculature after cast realized with two control and two n-3 LCPUFA tumors. * *p* < 0.05 (compared to control group, Mann–Whitney test). Magnification factor: 1.5×.

Vasculature quantification with CEUS was performed as soon as the mammary tumors of each nutritional group reached 2 cm² (W0, before docetaxel injection). The quantification of the CEUS signal, taking into account all contrast pixels within tumors, allowed us to show the global pixel density (GPD), reflecting the global vascular density. These results are presented in Figure 1A,B.

Before docetaxel treatment, the global vascular density (GPD, overall histogram) was similar between both groups (85% (74–93) and 85% (75–95), median (interquartiles)) (Figure 1A, left panels and Figure 1B). The specific analysis, including the pixel intensity discrimination, allowed us to access the vascular network architecture and quantify large, medium and small vessels. We used a histogram to present the GPD subdivided into the three compartments: micro-, medium- and macrovasculature (median values, bar interquartiles) (Figure 1B).

A remodeling of the vascular architecture was observed in the n-3 LCPUFA group, with a microvascularization enrichment of ~15% (48 to 55%) and a decreased macrovascularization of ~80% (3 to 0.6%) compared to tumors obtained in rats fed a control diet (Figure 1B, right panels). In addition, two rats per nutritional group were perfused with polymer PU4ii mixed. Then, a three-dimensional X-ray scan acquisition was performed. In agreement with the CEUS signal experiment, a decrease in the number of large vessels was also visible in vascular casting of n-3 LCPUFA tumors compared to control tumors (Figure 1C).

### 2.2. The Microvasculature Proportion Remains Preponderant in n-3 LCPUFA Tumors During Docetaxel Treatment and Correlates with Tumor Regression

**Figure 2:**
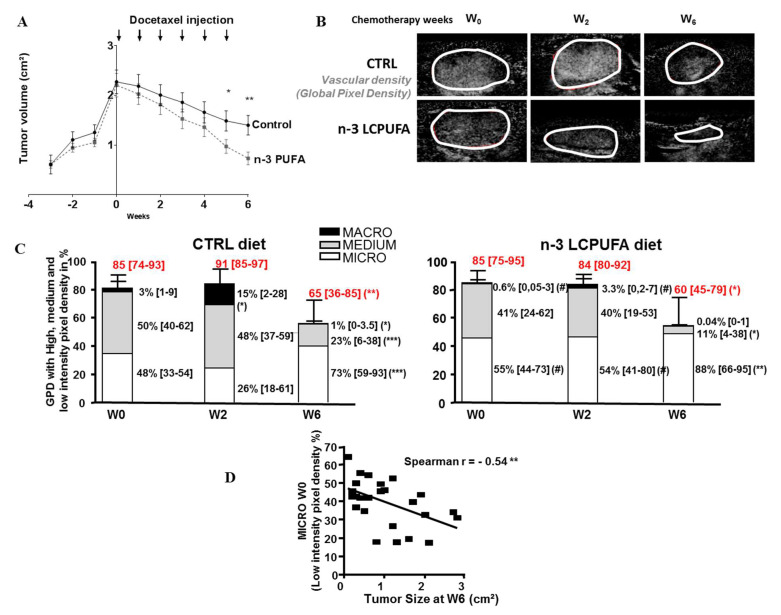
An n-3 LCPUFA-enriched diet maintains a microvascular compartment preponderance during docetaxel treatment. When tumors reached 2 cm^2^, docetaxel was injected (6mg/kg/week) every week for 6 weeks and tumor vascularization was quantified by CEUS methodology before (W0), or two weeks (W2) and six weeks (W6) after the beginning of docetaxel therapy (*n* = 14/nutritional group). Magnification factor: 1.5×. (**A**) Curves of tumors volumes (cm²) according to time (weeks). Tumor size was measured once a week for 6 weeks (mean ± SEM, *n* = 14) (**B**) Representative images obtained by CEUS showing GPD reflecting global vascular density within tumor of control or n-3 LCPUFA group (**C**) Histograms showing GPD (with its quantification in red) subdivided into three compartments: micro- (low intensity pixel density), medium- (medium intensity pixel density) and macro- (high intensity pixel density) vascularization in control and n-3 LCPUFA groups at W0, W2 and W6 (bar and values on the right: median and interquartile range, 25–75th percentile). * *p* < 0.05, *** *p* < 0.001 (compared to W0 in the same dietary group, paired t-test); # *p* < 0.05 (compared to control nutritional group at the corresponding time points, Mann–Whitney test). (**D**) Correlation between microvascularization measured before chemotherapy at W0 (%) and tumor size at the end of the chemotherapeutic treatment (W6). Points represent individual data (Spearman test, ** *p* < 0.001).

When tumor reached 2 cm^2^ (W0), docetaxel was injected once a week for 6 weeks, and the evolution of the different vascular compartments was followed by CEUS after 2 and 6 weeks of chemotherapy (W2 and W6). As expected and as already described in a previous study [12], in this new study, n-3 LCPUFA improved the efficacy of docetaxel by increasing tumor regression after 6 weeks of chemotherapy. We observed that tumor regression under docetaxel chemotherapy (between W0 and W6) reached 70% in the n-3 LCPUFA group compared to 40% in the control nutritional group (Figure 2A).

Figure 2B shows the evolution of the global vascular density (GPD, represented by all pixels with contrast signal) in each nutritional group before (W0), 2 weeks (W2), or 6 weeks (W6) after the beginning of the treatment. All CEUS results obtained for the two nutritional groups were compiled in Figure 2C. The global vascular densities (GPD values, overall histogram) were unchanged after 2 weeks of chemotherapy (W2) compared to W0. However, important differences in the tumor vascular architecture were observed at W2 in the control group, with an increased macrovascularization at W2 (a fivefold median change compared to W0) (Figure 2C). Interestingly, this effect was not observed in the n-3 LCPUFA tumor group in which microvascularization remained preponderant at W2. It represented 54% of the vasculature in the n-3 LCPUFA group and 26% in the control group (median values, *p* < 0.01). At W6, docetaxel induced an anti-angiogenic effect in the control diet group with a GPD reduction by 23% compared to W0, and a decrease in the levels of macro and medium compartments (median changes were threefold and twofold, respectively). Similar results were observed in n-3 LCPUFA-tumors. However, the effect of docetaxel on the vascular tree reorganization tended to be more important in n-3 LCPUFA tumors. The ratio of micro, medium, and macro-vessels was 73, 23, and 1% in the control group compared to 88, 11, and 0.04% in the n-3 LCPUFA group, respectively.

We tested whether the efficacy of docetaxel was enhanced in tumors with the highest density of microvasculature at the beginning of treatment (Figure 2D). A significant negative correlation was observed between the tumor microvasculature evaluated at W0 and tumor size measured after six cycles of chemotherapy (W6) (r² = −0.54, *p* < 0.01, Figure 2D). These data indicate that, in tumors with a high microvasculature density, regression is more efficient than in tumors with a reduced microvascularization density. Taken together, these experiments suggest a better distribution of the anticancer drug in tumors from rats fed an n-3 LCPUFA-enriched diet.

### 2.3. Epiregulin and Amphiregulin are New Molecular Targets of the n-3 LCPUFA-Enriched Diet, and Their mRNA Levels Correlate With Docetaxel Efficacy

Some of the effects mediated by n-3 LCPUFA on tumor vascular remodeling are similar to those reported following anti-VEGF therapy [2]. In the presented study, whereas plasma levels of VEGFα were decreased after six cycles of docetaxel chemotherapy compared to untreated tumors (Figure S1), no difference was observed between the control and the n-3 LCPUFA group. This finding indicates that the effect of n-3 LCPUFA on vascularization cannot be directly attributed to a change in plasma VEGFα levels.

To identify other potential molecular effectors of the n-3 LCPUFA-enriched diet, we used an angiogenesis PCR arrays (SuperArray Bioscience). This array includes 84 genes that have been involved in the modulation of angiogenesis (growth factors and receptors, adhesion molecules and matrix proteins, proteases and their inhibitors, cytokines and chemokines). The study was performed on tumors before (*n* = 4) or after six cycles of chemotherapy (*n* = 7) in both nutritional groups. Without docetaxel i.e., at W0 before chemotherapy starting, no statistical difference in term of mRNA expression levels between the two nutritional groups was observed. After completed chemotherapeutic treatment (W6), the levels of gene up- or downregulated in the n-3 LCPUFA group versus the control nutritional group are presented in Table S1. It can be observed that only VEGFβ among VEGF family members was increased 1.9-fold in n-3 LCPUFA tumors (*p* < 0.05). In addition, the selection of genes with a fold change greater than two included epiregulin (EREG, an epithelial growth factor (EGF) family member), matrix metallopeptidase 3 (MMP3), and tumor necrosis factor member 2 (TNFα). EREG and MMP3 were downregulated 6- (*p* < 0.01) and threefold (*p* < 0.05), respectively, in the n-3 LCPUFA group and TNFα was upregulated threefold (*p* < 0.05). Contrary to EREG, epithelial growth factor (EGF) expression levels were not modified in the two nutritional groups (Table S1).

**Figure 3:**
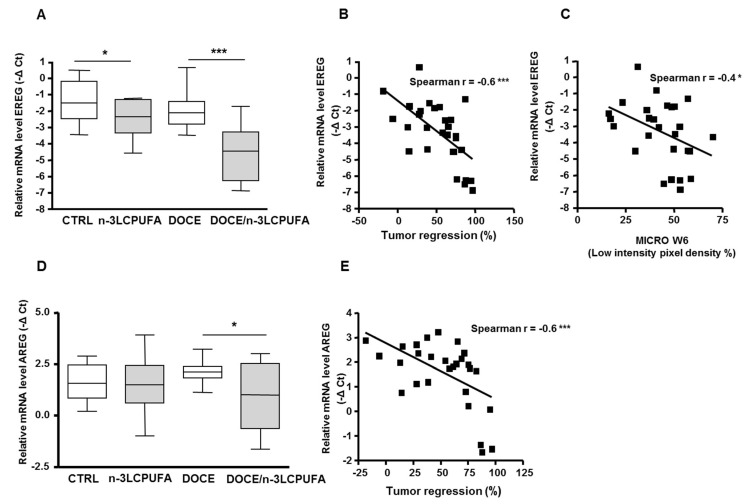
Regulation by n-3 LCPUFA of tumor epiregulin (EREG) and amphiregulin (AREG) mRNA expression levels is associated to tumor regression and microvascular compartment quantification. (**A**,**D**) EREG and AREG mRNA levels in mammary tumors in the two nutritional groups before (W0) and after docetaxel treatment (W6). (*n* = 14/nutritional group at W0 and *n* = 14/nutritional group at W6). qPCR results were expressed in -ΔCt. Lines are median values, * *p* < 0.05, *** *p* < 0.001, Man Whitney test. (**B**) Correlation between EREG mRNA levels at W6 and response to chemotherapy (expressed as percent of tumor regression). Points represent individual data (Spearman test, *** *p* < 0.001). (**C**) Correlation between EREG mRNA levels and microvasculature at W6. Points represent individual data (Spearman test, * *p* < 0.05, *** *p* < 0.001). (**E**) Correlation between AREG mRNA levels and microvasculature at W6. Points represent individual data (Spearman test, *** *p* < 0.001).

A qPCR analysis of these selected genes was extended to a larger number of tumors (*n* = 14 per nutritional group). With this more exhaustive analysis, the statistically significant differences obtained with VEGFβ, MMP3, and TNFα were not confirmed. In contrast, epiregulin (EREG) expression levels were confirmed as being clearly reduced ~4.5-fold in n-3 LCPUFA tumors compared to control tumors at W6 (*n* = 14, *p* < 0.001) (Figure 3A). In tumors without docetaxel, EREG expression levels were decreased twofold in n-3 LCPUFA tumors (*n* = 14, *p* < 0.05) (Figure 3A).

Correlations between EREG mRNA levels at W6 with tumor regression or vascular parameters were also examined. A significant negative correlation was identified between EREG expression and tumor regression (r = −0.6, *p* < 0.001, Figure 3B). Among the vascular parameters measured with CEUS at W6, EREG expression was negatively correlated with microvascularization (r = −0.4, *p* < 0.05) (Figure 3C). Taken together, these data suggested that a reduction in EREG mRNA expression levels within tumors was associated with an increased microvascular density and a better response to docetaxel treatment.

Gene transcription analysis was also performed with other EGF family members. No significant change in mRNA levels was detected for heparin-bound EGF (HB-EGF), β cellulin, tumor growth factor TGFα and neuregulin1 (NRG1) (Figure S2). Regarding the mRNA expression levels of amphiregulin (AREG), transcription levels of this gene were not affected by the diet alone, but mRNA expression levels of AREG were significantly downregulated (twofold) in docetaxel/n-3 LCPUFA tumors compared with docetaxel/control tumors (*p* < 0.05) (Figure 3D). As with EREG, a significant negative correlation was observed between AREG mRNA expression levels and tumor regression (r² = −0.6, *p* < 0.001, Figure 3E). However, only a modest correlation was found between AREG mRNA expression levels and microvasculature density (r = −0.3, *p* = 0.06).

## 3. Discussion

In the present study, the CEUS approach allowed us to demonstrate that n-3 LCPUFA can induce a remodeling of the tumor vascular architecture in favor of a microvasculature. This vascular compartment has been physiologically described as the most adapted compartment for molecules exchanges between the vascular bed and the interstitial fluid of tumors. In a healthy vascular tree, only microvessels (capillaries) display optimal parameters that favor pressure gradients (microvascular pressure, interstitial fluid pressure, and osmotic pressures) contributing to the exit of a therapeutic agent. Unlike what is observed in a normal vascular network, tumor vessels have been described as vasodilated and hyperpermeable [13,14]. These alterations can lead to increased interstitial fluid pressure and prevent adequate drug delivery [2,15]. In the present study, we show that, upon docetaxel treatment, the tumor vasculature with small caliber vessels remains preponderant in n-3 LCPUFA-tumors and is correlated with improved response to docetaxel chemotherapy. Mammary tumors with a high initial microvasculature (i.e., small caliber vessels) showed the best response to chemotherapy. This finding is in agreement with a better distribution of the anticancer drug. In a previous study, we have demonstrated that n-3 LCPUFA treatment reduces interstitial fluid pressure and improves tumor oxygenation and molecules extravasation [12]. The process of vascular normalization proposed by Jain et al. [16] may explain this observation. By restoring the balance between pro- and anti-angiogenic factors in the tumor microenvironment, antiangiogenic therapy restores a “normal” tumor vasculature by reducing vessel diameter. Accordingly, microvessels become preponderant at the expense of larger vessels. This type of vasculature allows the efficient delivery of cytotoxic agents into tumors and enhances chemotherapy efficacy [1,3]. Taken together, these data suggest that n-3 LCPUFA can “normalize” the tumor vasculature via architectural remodeling, which may improve functional vascular efficiency for drug delivery and tumor size regression.

Many studies have demonstrated that the application of moderate doses of anti-angiogenesis agents can normalize an abnormal tumor vascularization and improve blood perfusion and tumor therapy [2,17,18,19,20]. During vessel normalization, the architecture of the vasculature is largely restored, in a process that reduces the diameter of microvessels and prunes the most immature vasculature [21]. This effect creates a window of opportunity during which various concomitantly administered therapies are likely to be most effective [22,23].

With others, we have previously reported that n-3 LCPUFA displayed anti-angiogenic properties in rodent tumors [8,9] or in cultured endothelial cells [24,25]. Several mechanisms have been proposed to account for these antiangiogenic effects. n-3 LCPUFA can exert their effects on pathways regulating immune and inflammatory cells that participate in angiogenesis via the modulation of eicosanoid production and the production of anti-inflammatory n-3 PUFA-derived mediators (e.g., neuroprotectins and resolvins) [26,27,28]. In these conditions, a decrease in VEGF and/or its receptor levels has been reported in endothelial cells and colon cancer cells [25,29]. We have also shown a decreased in endothelial NO synthase activity with n-3 LCPUFA treatment and, subsequently, the production of NO, which is well known for its proangiogenic properties [12]. Other anti-angiogenic effects of DHA have been reported, and they may be mediated through secretion of exosome and their microRNA content [30].

Two new clinically relevant effectors of n-3 LCPUFA have been identified in the present study. Whereas the n-3 LCPUFA diet decreased EREG transcription levels in tumors, the combination of docetaxel with n-3 LCPUFA potentiated this downregulation. The docetaxel/n-3 LCPUFA combination was required to reduce AREG mRNA levels. The pro-oncogenic activities of AREG and EREG have been examined in previous studies of lung, breast, colorectal, and prostate carcinoma [31,32]. Importantly, the role of AREG and EREG in the regulation of tumorigenesis was underlined. These identified were self-sufficiency in the generation of growth signals, metastasis, angiogenesis, and resistance to various chemotherapeutic agents [31,32,33,34]. These two effectors are also clinically relevant since their mRNA levels in primary tumors can predict the outcome of metastatic colorectal cancer [35]. In two pre-clinical studies, AREG/EREG were not identified as regulated targets by docetaxel treatment [36,37]. However, AREG expression levels have been associated with disease progression of breast cancer or head and neck squamous cell carcinoma in patients receiving a combination treatment of anti-EGFRs and docetaxel [38,39]. The present study shows that EREG mRNA levels are inversely correlated with the mammary tumor microvascular density. This finding suggests a potential role for EREG in the vascular effects mediated by n-3 LCPUFA. This hypothesis is consistent with the previously described roles of EREG in angiogenesis [31].

Besides the effect of n-3 PUFAs in cancer, epidemiological and clinical studies have provided evidence, which suggest that n-3 LCPUFA and fish oils can offer cardiovascular disease protection. DHA plays an important role in the regulation of vascular endothelial cell function. Consumption of n-3 PUFAs, and in particular DHA, has been associated with improved endothelium function and prevention of cardiovascular disease via its effects on endothelial metabolism, inflammation, thrombosis, and arrhythmia [40].

In conclusion, this study demonstrates that dietary n-3 LCPUFA can influence vascular remodeling in mammary tumors. We also demonstrated the ability of the n-3 LCPUFA/docetaxel combination to downregulate EREG and AREG, which are two newly identified effectors with clinical relevance. Despite major clinical advances provided by cytotoxic, hormonal and targeted therapies, the median survival after diagnosis of metastatic breast cancer with visceral organ invasion remains below 2 years. The addition of a dietary strategy using n-3 LCPUFA may therefore be relevant to complement the clinical strategy to improve drug efficacy in mammary tumors and decrease anticancer drug resistance. The present preclinical study strengthens the findings of three previously published Phase II clinical trial using n-3 LCPUFA for patients treated for breast, lung, or colon cancers [7,41,42]. In addition, it reinforces the rationale for the organization of a phase III clinical trial, which would test the effects of n-3 LCPUFA supplementation on the efficacy of conventional cancer treatment.

## 4. Materials and Methods

### 4.1. In Vivo Study

**Figure 4:**
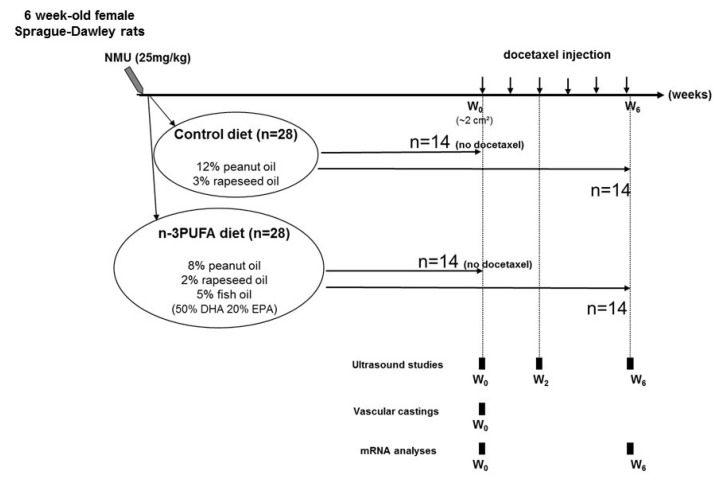
Diagram summarizing the in vivo experimental design. Mammary carcinogenesis was initiated with a single dose of N-methylnitrosourea. Three days later, an experimental diet was provided until the end of the study. Rats were divided into a control (*n* = 28) and an n–3 LCPUFA diet group (*n* = 28). When tumors reached 2 cm^3^ (W0), rats were treated with docetaxel using one injection/week (arrow) (*n* = 14/nutritional group) or euthanized (*n* = 14/nutritional group). Functional vascular and mRNA analyses were performed at the indicated time points before (W0), 2 (W2), or 6 weeks (W6) after the start of docetaxel treatment.

Animal protocols used for these studies were approved by the Val de Loire Animal Ethics Committee (approved on 13 January 2006, approval code: CL2006-001). Experimental carcinogenesis induction and diets have previously been described by Kornfeld et al. [12]. The experimental design is summarized in Figure 4.

Mammary carcinogenesis was induced in 48 days-old female Sprague-Dawley rats by injection of a single dose of 25 mg/kg n-methyl-n-nitrosourea (NMU). Three days after induction, an experimental diet was provided until the end of the study. Rats from the control group (*n* = 28) were fed a diet containing peanut oil (12%) and rapeseed oil (3%) (%, g/100 g of diet). Rats from the n-3 LCPUFA group (*n* = 28) were fed a diet containing peanut oil (8%), rapeseed oil (2%), and fish oil (5%). Importantly, the n-3 LCPUFA-enriched diet contained 2.5% DHA and 1% EPA. When tumors reached 2 cm^3^ (week 0), 14 out of 28 rats from each group were treated intraperitoneally once a week with 6 mg/kg/week docetaxel (Taxotere^®^, Sanofi Aventis, France) for 6 weeks. Rats were examined weekly and tumor area was calculated using the ellipse formula: tumor area= π (Ø1 * Ø2)/4 where Ø1 and Ø2 are the two largest diameters. The beginning of the chemotherapy was set as the reference, and tumor regression was calculated as the percentage of tumor area variation between tumor size before docetaxel treatment and at the end of treatment. qPCR analysis was performed on tumors without docetaxel treatment or after 6 chemotherapeutic cycles (week 6). Animals were individually monitored twice weekly to follow their health status, record their weight, and assess global aspect and activity level. During this experiment, no animals became severely ill or died at any time prior to the experimental endpoint. Rats were euthanized at the endpoint with a pentobarbital overdose (150 mg/kg, intraperitoneal).

### 4.2. Functional Vascularization and Quantification of Micro, Medium and Macrovascularization

We used Contrast-Enhanced Ultrasound (CEUS) to investigate Tumor vascularization. Acquisition of CEUS data was performed with a dedicated CnTI™ technology (Contrast Tuned Imaging, Esaote, Genoa, Italy) using a Technos scanner equipped with a 5–10 MHz probe (LA 532, Esaote, Italy). Microbubbles from SonoVue^®^ (Bracco Imaging SpA, Milano, Italy) were used as a contrast agent (0.3 mL, 45 µg/mL, intravenous bolus injection) only to perfuse functional blood vessels [43]. Echographic explorations were performed under 2.5% isoflurane anesthesia.

The strongly echogenic microbubbles contained sulfur hexafluoride, which is a gas encapsulated within a phospholipid shell [44]. With diameters of up to 10 µm, they remained in the vascular bed and were not appropriate to evaluate vascular leakage. The specific CEUS analysis used for quantification of micro, medium and macrovascularization has previously been described [4]. Briefly, using the Visilog 6 software version 1.52 (Noesis, Courbaboeuf, France), CEUS signal from a video clip was analyzed within the tumors after defining the tumor as the region of interest (ROI). Two frames (one before SonoVue^®^ arrival and one at the peak of contrast enhancement) were selected from the CEUS video clip. Pixels from the first frame (background tissue signal) were subtracted from the peak frame. All contrast-enhanced pixels in the ROI defined the global pixel density (GPD) corresponding to a global vascular density. Larger vessels contained more contrast agent than the smaller ones, leading to a scaled intensity of contrast enhancement. Using the MATLAB^®^ software (MathWorks, Natick MA, USA), the CEUS signal was analyzed to discriminate between pixels according to their contrast intensity and allowed us to determine high- (intensity 170–255), medium- (intensity 85–170) and low-intensity pixels (intensity 1–85). The lowest intensity pixel density (from 1 to 85) was used to quantify microvascularization. While the medium contrast-enhanced pixel density (85–170) allowed us to calculate medium vascularization, the brightest pixels density permitted to quantify macrovascularization. To report the different vascular tree proportions, high-, medium- and low-intensity pixels densities were finally expressed as a percentage of the ROI. Importantly, we have previously validated this CEUS method by standard immunohistological analysis [4].

### 4.3. Casting of the Tumor Vasculature

After anesthesia, polymer PU4ii (VasQtec, Zürich, Switzerland) mixed with lipiodol (X-ray contrast agent, Guerbet, Villepinte, France) was used to perfused rats (CERB, Baugy, France). After X-ray scan acquisition (Imaging Center, CIPA, Orléans, France), a modified Feldkamp cone-beam reconstruction algorithm with 50 µm^3^ isotropic voxels (CT 120 and Micro View Analysis; GE Healthcare, Buc, France) was used to reconstruct the raw data.

### 4.4. Oligonucleotide PCR Arrays and Real-Time Quantitative PCR Analysis

Total RNA from tumor samples were extracted with TRIzol reagent (Invitrogen, Life Technologies, Courtaboeuf, France). RNA was reverse transcribed using a cDNA synthesis kit (“Ready-to-go”, GE Healthcare, Buc, France) following the manufacturer’s instructions. cDNA was used for real-time PCR analyses with the Angiogenesis RT² ProfilerTM PCR Array (SuperArray Bioscience Corporation, Frederick, MD, USA). The 84 genes of the PCR array were involved in the modulation of angiogenesis (gene list provided in supplementary data 2). Real-time qPCR was carried out with MyiQ thermocycler (Biorad, Marnes-la-Coquette, France) using Platinum^®^ SYBR^®^ Green qPCR SuperMix-UDG kit (Invitrogen, Life Technologies, Courtaboeuf, France). The thermal cycling parameters were as follows: 10 min at 95 °C, followed by 40 cycles of 15 s at 95 °C, 30 s at 60 °C, and 30 s at 72 °C.

Complementary analyses were performed using the rat primer sequences for EREG (epiregulin), AREG (amphiregulin), β cellulin, heparin-binding EGF, tumor growth factor α neuregulin1 and reference gene hypoxanthine phosphoribosyltransferase 1 (HPRT1) (Sigma-Aldrich, Saint-Quentin Fallavier, France) were as follows: EREG forward, 5′-CCGTGATTCTCGTTTTCCTC-3′ and reverse 5′-TTTACTTTTGCGATTTCTGTACCA-3′; AREG forward, 5′-GGTGAATGCAGATACATCGAGA-3′ and reverse 5′-CGTTCGCCAAAGTAATCCTG-3′; β cellulin for 5′-TGAAACCAATGGCTCTCTTTG and rev 5′-CGATTTCTGTCTAGGGGTGGT-3′; heparin-binding EGF: for 5′-TGGGGCTTCTCATGTTTAGG-3′ and rev 5′-CATGCCCAACTTCACTTTCTC-3′, tumor growth factor α: for 5′-TTGCTGCCACTCAGAAACAG-3′ and rev 5′-ATCTGCCACAGTCCACCTG-3′, neuregulin1: for 5′-CTGCTAGCCCCTTGAGGAT-3′ and rev 5′-GCTCGTACTCTTGGGTCGTTT-3′, HPRT1 for 5′-GAC CGG TTC TGT CAT GTC G-3′ and rev 5′-ACC TGG TTC ATC ATC ACT AAT CAC-3′.

### 4.5. Statistical Analyses

Statistical analyses were carried out using the GraphPad Prism 4 software (La Jolla, CA, USA). Mann–Whitney tests and paired t tests were used. Spearman test was used for correlation. *P*-values below 0.05 were considered statistically significant.

## 5. Conclusions

In conclusion, this study demonstrates that dietary n-3 LCPUFA can influence vascular remodeling in mammary tumors. We also demonstrated the ability of n-3 LCPUFA to downregulate EREG and AREG, which are two newly identified effectors with clinical relevance.

## Supplementary Materials

Supplementary materials can be found at https://www.mdpi.com/1422-0067/21/14/4965/s1. Figure S1. Plasma VEGF-A levels decrease during docetaxel treatment and are unaffected by n-3 LCPUFA diet. Plasma VEGF-A levels were determined in rats from the two nutritional groups before (W0) and after docetaxel treatment (W6) (12 < *n* < 14 per group) using a rat VEGF elisa kit (RayBiotech, Norcross, GA). Points represent individual data ** *p* < 0.01 Mann–Whitney test. Figure S2. Heparin-bound EGF, β cellulin, tumor growth factor α and neuregulin 1 mRNA are not regulated in tumors by an n-3 LCPUFA diet. When tumors reached 2 cm^2^, docetaxel was injected for 6 weeks, and qPCR analyses were performed before (W0) or after 6 weeks (W6) of docetaxel therapy (*n* = 14/nutritional group). mRNA levels of heparin-bound EGF (HB-EGF) (A), β cellulin (B), tumor growth factor α (C) and neuregulin 1 (D) in the two nutritional groups before or after docetaxel treatment (W6). mRNA levels of each gene were expressed relatively to the HPRT1 housekeeping gene (-∆Ct). Lines as median values and box, interquartile range, 25–75th percentile. Table S1. Gene list of targets analyzed in the angiogenesis PCR arrays and regulation of gene expression in n-3 LCPUFA tumors after docetaxel treatment. When tumors reached 2 cm^2^, docetaxel was injected and qPCR analysis was performed after 6 weeks (W6) of docetaxel therapy (*n* = 7/nutritional group). cDNA were used for real-time PCR analyses with the Angiogenesis RT² ProfilerTM PCR Array (SuperArray Bioscience Corporation, Frederick, MD, USA). The 84 genes in this PCR array are involved in the modulation of angiogenesis. A positive fold change indicates upregulation, whereas a negative fold change indicates downregulation of the corresponding mRNA in n-3 LCPUFA tumors compared to control tumors. (*n* = 7/group). Genes highlighted in bold correspond to those with mRNA levels showing differences of more than twofold change between the two nutritional groups (t-test).

## Abbreviations

AREG	Amphiregulin
CEUS	Contrast-Enhanced Ultrasound
DHA	Docosahexaenoic Acid
EPA	Eicosapentaenoic Acid
EREG	Epiregulin
GPD	Global pixel density
n-3 LCPUFA	n-3 Long Chain Polyunsaturated Fatty Acids
NMU	N-methylnitrosourea
VEGF	Vascular endothelial growth factor

## References

[1] Fukumura D., Jain R.K. (2007). Tumor microvasculature and microenvironment: Targets for anti-angiogenesis and normalization. Microvasc. Res..

[2] Tong R.T., Boucher Y., Kozin S.V., Winkler F., Hicklin D.J., Jain R.K. (2004). Vascular Normalization by Vascular Endothelial Growth Factor Receptor 2 Blockade Induces a Pressure Gradient Across the Vasculature and Improves Drug Penetration in Tumors. Cancer Res..

[3] Jain R.K. (2005). Normalization of Tumor Vasculature: An Emerging Concept in Antiangiogenic Therapy. Science.

[4] Maheo K., Chevalier S., Vibet S., Bougnoux P., Richard S., Sérrière S., Bleuzen A., Tranquart F., Goupille C. (2012). Non-invasive quantification of tumor vascular architecture during docetaxel-chemotherapy. Breast Cancer Res. Treat..

[5] Biondo P.D., Brindley D.N., Sawyer M.B., Field C.J. (2008). The potential for treatment with dietary long-chain polyunsaturated n-3 fatty acids during chemotherapy. J. Nutr. Biochem..

[6] Calviello G., Serini S., Piccioni E., Pessina G. (2009). Antineoplastic Effects of N-3 Polyunsaturated Fatty Acids in Combination With Drugs and Radiotherapy: Preventive and Therapeutic Strategies. Nutr. Cancer.

[7] Bougnoux P., Hajjaji N., Maheo K., Couet C., Chevalier S. (2011). Fatty acids and breast cancer: Sensitization to treatments and prevention of metastatic re-growth. Prog. Lipid Res..

[8] Colas S., Mahéo K., Denis F., Goupille C., Hoinard C., Champeroux P., Tranquart F., Bougnoux P. (2006). Sensitization by Dietary Docosahexaenoic Acid of Rat Mammary Carcinoma to Anthracycline: A Role for Tumor Vascularization. Clin. Cancer Res..

[9] Rose D.P., Connolly J.M. (1999). Antiangiogenicity of docosahexaenoic acid and its role in the suppression of breast cancer cell growth in nude mice. Int. J. Oncol..

[10] Szymczak M., Murray M., Petrović N. (2008). Modulation of angiogenesis by ω-3 polyunsaturated fatty acids is mediated by cyclooxygenases. Blood.

[11] Zhang G., Panigrahy D., Mahakian L.M., Yang J., Liu J.-Y., Lee K.S.S., Wettersten H.I., Ulu A., Hu X., Tam S. (2013). Epoxy metabolites of docosahexaenoic acid (DHA) inhibit angiogenesis, tumor growth, and metastasis. Proc. Natl. Acad. Sci. USA.

[12] Kornfeld S., Goupille C., Vibet S., Chevalier S., Pinet A., Lebeau J., Tranquart F., Bougnoux P., Martel E., Maurin A. (2012). Reducing endothelial NOS activation and interstitial fluid pressure with n-3 PUFA offset tumor chemoresistance. Carcinogenesis.

[13] Jain R.K. (2003). Molecular regulation of vessel maturation. Nat. Med..

[14] Baluk P., Hashizume H., McDonald D.M. (2005). Cellular abnormalities of blood vessels as targets in cancer. Curr. Opin. Genet. Dev..

[15] Heldin C.-H., Rubin K., Pietras K., Östman A. (2004). High interstitial fluid pressure—An obstacle in cancer therapy. Nat. Rev. Cancer.

[16] Goel S., Wong H.-K., Jain R.K. (2011). Vascular Normalization as a Therapeutic Strategy for Malignant and Nonmalignant Disease. Cold Spring Harb. Perspect. Med..

[17] Huang Y., Stylianopoulos T., Duda D.G., Fukumura D., Jain R.K. (2013). Benefits of Vascular Normalization Are Dose and Time Dependent. Cancer Res..

[18] Chatterjee S., Wieczorek C., Schöttle J., Siobal M., Hinze Y., Franz T., Florin A., Adamczak J., Heukamp L., Neumaier B. (2014). Transient Antiangiogenic Treatment Improves Delivery of Cytotoxic Compounds and Therapeutic Outcome in Lung Cancer. Cancer Res..

[19] Weiss A., Bonvin D., Berndsen R.H., Scherrer E., Wong T.J., Dyson P.J., Griffioen A.W., Nowak-Sliwinska P. (2015). Angiostatic treatment prior to chemo- or photodynamic therapy improves anti-tumor efficacy. Sci. Rep..

[20] Claes A., Wesseling P., Jeuken J., Maass C., Heerschap A., Leenders W.P.J. (2008). Antiangiogenic compounds interfere with chemotherapy of brain tumors due to vessel normalization. Mol. Cancer Ther..

[21] Carmeliet P., Jain R.K. (2011). Principles and mechanisms of vessel normalization for cancer and other angiogenic diseases. Nat. Rev. Drug Discov..

[22] Winkler F., Kozin S.V., Tong R.T., Chae S.-S., Booth M.F., Garkavtsev I., Xu L., Hicklin D.J., Fukumura D., Di Tomaso E. (2004). Kinetics of vascular normalization by VEGFR2 blockade governs brain tumor response to radiation. Cancer Cell.

[23] Koyama S., Matsunaga S., Imanishi M., Maekawa Y., Kitano H., Takeuchi H., Tomita S. (2017). Tumour blood vessel normalisation by prolyl hydroxylase inhibitor repaired sensitivity to chemotherapy in a tumour mouse model. Sci. Rep..

[24] Tsuzuki T., Shibata A., Kawakami Y., Nakagaya K., Miyazawa T. (2007). Anti-Angiogenic Effects of Conjugated Docosahexaenoic Acidin Vitroandin Vivo. Biosci. Biotechnol. Biochem..

[25] Tsuji M., Murota S.-I., Morita I. (2003). Docosapentaenoic acid (22:5, n-3) suppressed tube-forming activity in endothelial cells induced by vascular endothelial growth factor. Prostaglandins, Leukot. Essent. Fat. Acids.

[26] Calder P.C. (2006). Polyunsaturated fatty acids and inflammation. Prostaglandins, Leukot. Essent. Fat. Acids.

[27] Calder P.C. (2013). n-3 Fatty acids, inflammation and immunity: New mechanisms to explain old actions. Proc. Nutr. Soc..

[28] Connor K.M., SanGiovanni J.P., Löfqvist C., Aderman C.M., Chen J., Higuchi A., Hong S., Pravda E.A., Majchrzak S., Carper D. (2007). Increased dietary intake of ω-3-polyunsaturated fatty acids reduces pathological retinal angiogenesis. Nat. Med..

[29] Calviello G., Di Nicuolo F., Gragnoli S., Piccioni E., Serini S., Maggiano N., Tringali G., Navarra P., Ranelletti F.O., Palozza P. (2004). n-3 PUFAs reduce VEGF expression in human colon cancer cells modulating the COX-2/PGE2 induced ERK-1 and -2 and HIF-1alpha induction pathway. Carcinogenesis.

[30] Hannafon B.N., Carpenter K.J., Berry W.R., Janknecht R., Dooley W., Ding W.-Q. (2016). Exosome-mediated microRNA signaling from breast cancer cells is altered by the anti-angiogenesis agent docosahexaenoic acid (DHA). Mol. Cancer.

[31] Riese D., Cullum R.L. (2014). Epiregulin: Roles in normal physiology and cancer. Semin. Cell Dev. Boil..

[32] Busser B., Sancey L., Brambilla E., Coll J.-L., Hurbin A. (2011). The multiple roles of amphiregulin in human cancer. Biochim. Biophys. Acta (BBA)—Bioenerg..

[33] Farooqui M., Bohrer L.R., Brady N.J., Chuntova P., Kemp S.E., Wardwell C.T., Nelson A.C., Schwertfeger K.L. (2015). Epiregulin contributes to breast tumorigenesis through regulating matrix metalloproteinase 1 and promoting cell survival. Mol. Cancer.

[34] Eckstein N., Servan K., Girard L., Cai D., Von Jonquieres G., Jaehde U., Kassack M.U., Gazdar A.F., Minna J.D., Royer H.-D. (2008). Epidermal growth factor receptor pathway analysis identifies amphiregulin as a key factor for cisplatin resistance of human breast cancer cells. J. Boil. Chem..

[35] Jacobs B., De Roock W., Piessevaux H., Van Oirbeek R., Biesmans B., De Schutter J., Fieuws S., Vandesompele J., Peeters M., Van Laethem J.-L. (2009). Amphiregulin and Epiregulin mRNA Expression in Primary Tumors Predicts Outcome in Metastatic Colorectal Cancer Treated With Cetuximab. J. Clin. Oncol..

[36] Nagai M.A., Dos Santos M.L., Gimenes K.P., Silva W.A. (2009). Transcriptome changes induced by docetaxel in human mammary cell lines expressing different levels of ERBB2. Int. J. Mol. Med..

[37] Hernández-Vargas H., Palacios J., Moreno-Bueno G. (2007). Molecular profiling of docetaxel cytotoxicity in breast cancer cells: Uncoupling of aberrant mitosis and apoptosis. Oncogene.

[38] Kim J.-W., Kim D.K., Min A., Lee K.-H., Nam H.-J., Kim J.H., Kim J.-S., Kim T.Y., Im S.-A., Park I.A. (2016). Amphiregulin confers trastuzumab resistance via AKT and ERK activation in HER2-positive breast cancer. J. Cancer Res. Clin. Oncol..

[39] Tinhofer I., Klinghammer K., Weichert W., Stenzinger A., Gauler T., Budach V., Keilholz U., Knödler M. (2011). Expression of Amphiregulin and EGFRvIII Affect Outcome of Patients with Squamous Cell Carcinoma of the Head and Neck Receiving Cetuximab-Docetaxel Treatment. Clin. Cancer Res..

[40] Yamagata K. (2017). Docosahexaenoic acid regulates vascular endothelial cell function and prevents cardiovascular disease. Lipids Heal. Dis..

[41] Murphy R.A., Mourtzakis M., Chu Q.S.C., Baracos V.E., Reiman T., Mazurak V.C. (2011). Supplementation with fish oil increases first-line chemotherapy efficacy in patients with advanced nonsmall cell lung cancer. Cancer.

[42] Cockbain A.J., Toogood G., Hull M.A. (2011). Omega-3 polyunsaturated fatty acids for the treatment and prevention of colorectal cancer. Gut.

[43] Broillet A., Hantson J., Ruegg C., Messager T., Schneider M. (2005). Assessment of microvascular perfusion changes in a rat breast tumor model using SonoVue to monitor the effects of different anti-angiogenic therapies. Acad. Radiol..

[44] Schneider M. (1999). Characteristics of SonoVuetrade mark. Echocardiography.

