# Supplementary material for: EPA and DHA Fatty Acids Induce a Remodeling of Tumor Vasculature and Potentiate Docetaxel Activity
Source: Int J Mol Sci. 2020 Jul 14;21(14):4965. doi: 10.3390/ijms21144965 (PMC7404030; doi:10.3390/ijms21144965)
Supplement: Supplementary file 1 [file ijms-21-04965-s001.pdf]

Supplementary Materials

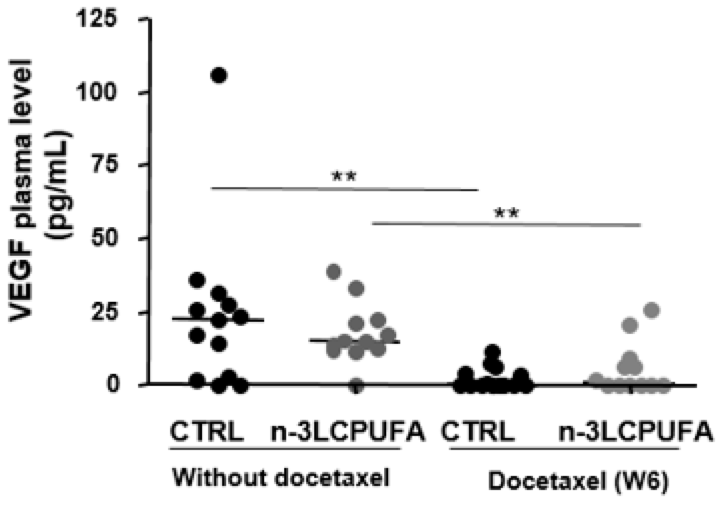

Figure S1. Plasma VEGF-A levels decrease during docetaxel treatment and are unaffected by n-3LCPUFA diet. Plasma VEGF-A levels were determined in rats from the two nutritional groups before (W0) and after docetaxel treatment (W6) ($12 < n < 14$ per group) using a rat VEGF elisa kit (RayBiotech, Norcross, GA). Points represent individual data ** $p < 0.01$ Mann–Whitney test.

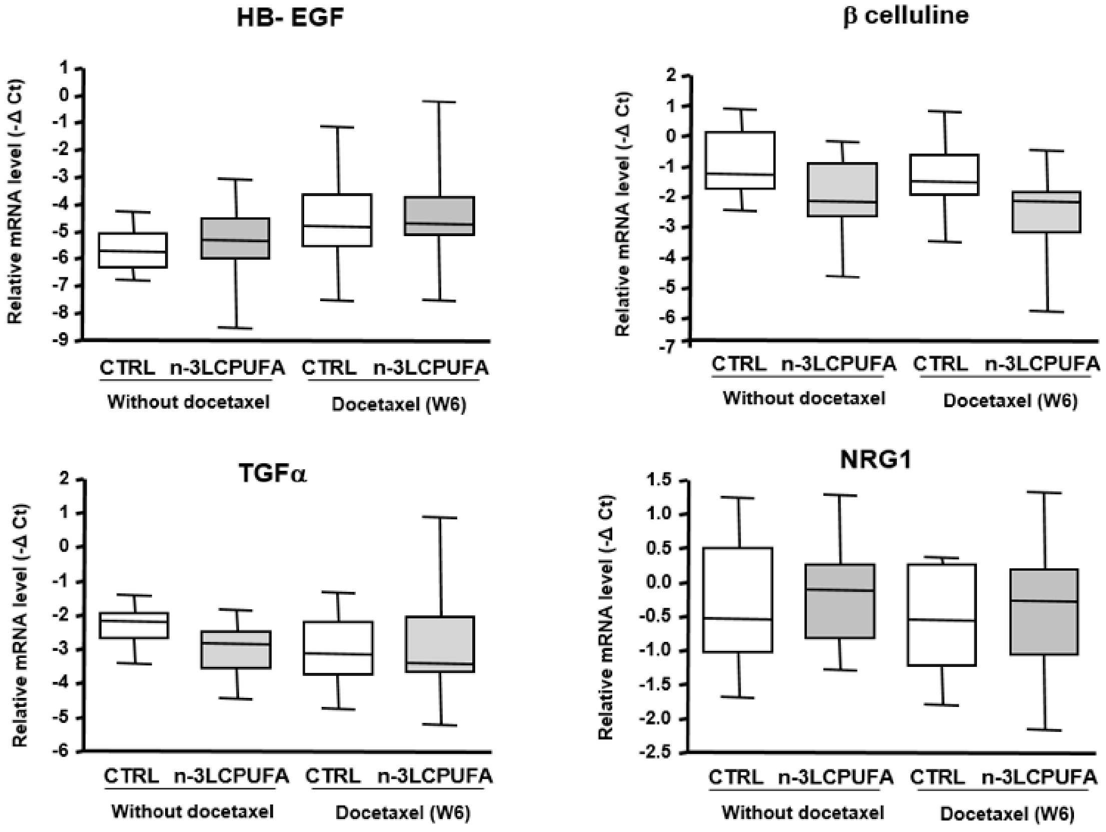

Figure S2. Heparin-bound EGF, β cellulin, tumor growth factor *α* and neuregulin 1 mRNA are not regulated in tumors by an n-3LCPUFA diet. When tumors reached 2 cm^2, docetaxel was injected for 6 weeks, and qPCR analyses were performed before (W0) or after 6 weeks (W6) of docetaxel therapy (n = 14/nutritional group). mRNA levels of heparin-bound EGF (HB-EGF) (**A**), β cellulin (**B**), tumor growth factor *α* (**C**) and neuregulin 1 (**D**) in the two nutritional groups before or after docetaxel treatment (W6). mRNA levels of each gene were expressed relatively to the HPRT1 housekeeping gene (-ΔCt). Lines as median values and box, interquartile range, 25–75th percentile.

Table S1. Gene list of targets analyzed in the angiogenesis PCR arrays and regulation of gene expression in n-3LCPUFA tumors after docetaxel treatment. When tumors reached 2 cm^2, docetaxel was injected and qPCR analysis was performed after 6 weeks (W6) of docetaxel therapy (n = 7/nutritional group). cDNA were used for real-time PCR analyses with the Angiogenesis RT^2 ProfilerTM PCR Array (SABiosciences). The 84 genes in this PCR array are involved in the modulation of angiogenesis. A positive fold change indicates up-regulation whereas a negative fold change indicates down-regulation of the corresponding mRNA in n-3LCPUFA tumors compared to control tumors. (n = 7/group). Genes highlighted in bold correspond to those with mRNA levels showing differences of more than 2-fold change between the 2 nutritional groups (t-test).

Symbol	Gene name	Fold Up- or Down-Regulation	t-TEST
			p value
Angpt2	Angiopoietin 2	2.16	0.3756
Akt1	V-akt murine thymoma viral oncogene homolog 1	1.78	0.0289
Angpt1	Angiopoietin 1	−1.18	0.5561
Anpep	Alanyl (membrane) aminopeptidase	1.69	0.5352
Bai1_predicted	Similar to Brain-specific angiogenesis inhibitor 1 precursor (LOC362931), mRNA	1.90	0.0404
Ccl2	Chemokine (C-C motif) ligand 2	−1.02	0.9799
Cdh5_predicted	Cadherin 5 (predicted)	1.52	0.4340

Col18a1	Collagen, type XVIII, alpha 1	−1.26	0.6348
Col4a3	Collagen, type IV, alpha 3	−3.53	0.0819
Ctgf	Connective tissue growth factor	1.07	0.8843
Cxcl1	Chemokine (C-X-C motif) ligand 1	−1.24	0.7312
Cxcl2	Chemokine (C-X-C motif) ligand 2	1.81	0.5694
Cxcl9	Chemokine (C-X-C motif) ligand 9	1.15	0.9043
Ecgf1	Endothelial cell growth factor 1 (platelet-derived)	1.15	0.5416
Edg1	Endothelial differentiation sphingolipid G-protein-coupled receptor 1	1.40	0.4984
Efna1	Ephrin A1	1.60	0.5404
Efna2	Ephrin A2	1.92	0.0646
Efna5	Ephrin A5	1.30	0.3729
Egf	Epidermal growth factor	2.06	0.0987
Eng	Endoglin	1.66	0.0944
Epas1	Endothelial PAS domain protein 1	1.80	0.1275
Ereg	Epiregulin	−6.18	0.0019
F2	Coagulation factor 2	−1.10	0.8435
Fgf1	Fibroblast growth factor 1	−2.14	0.4921
Fgf16	Fibroblast growth factor 16	1.89	0.3439
Fgf2	Fibroblast growth factor 2	1.72	0.1996
Fgf6	Fibroblast growth factor 6	1.59	0.0520
Fgfr3	Fibroblast growth factor receptor 3	1.26	0.7778
Figf	C-fos induced growth factor (vascular endothelial growth factor D)	−1.15	0.8079
Flt1	FMS-like tyrosine kinase 1	1.22	0.8092
Fn1	Fibronectin 1	−1.12	0.7910
Fzd5	Frizzled homolog 5 (Drosophila)	1.20	0.6217
Hgf	Hepatocyte growth factor	1.00	0.9964
Hif1a	Hypoxia inducible factor 1, alpha subunit	1.01	0.9474
Id1	Inhibitor of DNA binding 1, helix-loop-helix protein (splice variation)	−1.06	0.9043
Id3	Inhibitor of DNA binding 3, dominant negative helix-loop-helix protein	1.65	0.3728
Ifnb1	Interferon, beta 1	1.59	0.0520
Ifng	Interferon gamma	1.56	0.3019
Igf1	Insulin-like growth factor 1	1.01	0.9931
Il1b	Interleukin 1 beta	1.16	0.7178
Il6	Interleukin 6	−2.12	0.2142
Itga5	Integrin alpha 5	1.39	0.1351
Itgav_predicted	Integrin, alpha V (vitronectin receptor, alpha polypeptide, antigen CD51) (predicted)	−1.20	0.4721
Itgb3	Integrin beta 3	1.91	0.0933
Jag1	Jagged 1	1.05	0.8681
Kdr	Kinase insert domain protein receptor	1.42	0.4083
Lama5	Laminin, alpha 5	1.19	0.7167
Lect1	Leukocyte cell derived chemotaxin 1	1.08	0.8598
Lep	Leptin	1.08	0.9388
Mapk14	Mitogen activated protein kinase 14	1.02	0.9774
Mdk	Midkine	1.31	0.5223
Mmp19_predicted	Matrix metalloproteinase 19 (predicted)	−1.19	0.5645
Mmp2	Matrix metallopeptidase 2	−1.00	0.9944
Mmp3	Matrix metallopeptidase 3	−3.38	0.0362
Mmp9	Matrix metallopeptidase 9	2.77	0.1003
Npr1	Natriuretic peptide receptor 1	1.28	0.4819
Nrp1	Neuropilin 1	1.18	0.6640
Nrp2	Neuropilin 2	1.72	0.1285
Pdgfa	Platelet derived growth factor, alpha	2.02	0.0734
Pdgfb	Platelet derived growth factor, B polypeptide	1.18	0.9176

Pecam	Platelet/endothelial cell adhesion molecule	1.79	0.1559
Pgf	Placental growth factor	−1.03	0.9323
Plau	Plasminogen activator, urokinase	1.18	0.7891
Plg	Plasminogen	−1.49	0.4100
Ptgs1	Prostaglandin-endoperoxide synthase 1	−1.08	0.8337
Serpinb5	Serine (or cysteine) proteinase inhibitor, clade B, member 5	−1.63	0.2476
Serpinf1	Serine (or cysteine) proteinase inhibitor, clade F), member 1	1.17	0.8138
Sphk1	Sphingosine kinase 1	2.12	0.0582
Tbx4_predicted	T-box 4 (predicted)	−1.47	0.2583
Tek	Endothelial-specific receptor tyrosine kinase	1.60	0.3283
Tgfa	Transforming growth factor alpha	−1.39	0.5544
Tgfb1	Transforming growth factor, beta 1	1.76	0.5857
Tgfb2	Transforming growth factor, beta 2	1.76	0.0786
Tgfb3	Transforming growth factor, beta 3	1.49	0.7266
Tgfbr1	Transforming growth factor, beta receptor 1	1.30	0.4628
Thbs4	Thrombospondin 4	−2.77	0.1172
Timp1	Tissue inhibitor of metalloproteinase 1	−1.48	0.3264
Timp2	Tissue inhibitor of metalloproteinase 2	1.39	0.7986
Timp3	Tissue inhibitor of metalloproteinase 3 (Sorsby fundus dystrophy, psuedo inflammatory)	2.2	0.1151
Tnf	Tumor necrosis factor superfamily, member 2	3.63	0.0330
Vegfa	Vascular endothelial growth factor A	1.59	0.2566
Vegfb	Vascular endothelial growth factor B	1.92	0.033
Vegfc	Vascular endothelial growth factor C	−1.09	0.9391
Rplp1	Ribosomal protein, large, P1	1.09	0.5500
Hprt	Hypoxanthine guanine phosphoribosyl transferase	−1.15	0.5743
Rpl13a	Ribosomal protein L13A	1.02	0.8920
Ldha	Lactate dehydrogenase A	−1.29	0.4538
Actb	Actin, beta	1.51	0.0439
